# Systemic transplantation of adult multipotent stem cells prevents articular cartilage degeneration in a mouse model of accelerated ageing

**DOI:** 10.1186/s12979-021-00239-8

**Published:** 2021-06-07

**Authors:** Seth D. Thompson, Rajeswari Pichika, Richard L. Lieber, Mitra Lavasani

**Affiliations:** 1 Shirley Ryan Abilitylab (Formerly the Rehabilitation Institute of Chicago), 355 E. Erie St, IL 60611 Chicago, USA; 2 Department of Physical Medicine and Rehabilitation, Northwestern University, Chicago, USA; 3 Northwestern University Interdepartmental Neuroscience (NUIN) Graduate Program, Northwestern University, Chicago, USA

**Keywords:** Progeria, Accelerated ageing, Articular cartilage, Adult stem cells, Transplantation, Regenerative medicine

## Abstract

**Background:**

Osteoarthritis (OA) is one of the most prevalent joint diseases of advanced age and is a leading cause of disability worldwide. Ageing is a major risk factor for the articular cartilage (AC) degeneration that leads to OA, and the age-related decline in regenerative capacity accelerates OA progression. Here we demonstrate that systemic transplantation of a unique population of adult multipotent muscle-derived stem/progenitor cells (MDSPCs), isolated from young wild-type mice, into *Zmpste24*^*−/−*^ mice (a model of Hutchinson-Gilford progeria syndrome, a condition marked by accelerated ageing), prevents ageing-related homeostatic decline of AC.

**Results:**

MDSPC treatment inhibited expression of cartilage-degrading factors such as pro-inflammatory cytokines and extracellular matrix-proteinases, whereas pro-regenerative markers associated with cartilage mechanical support and tensile strength, cartilage resilience, chondrocyte proliferation and differentiation, and cartilage growth, were increased. Notably, MDSPC transplantation also increased the expression level of genes known for their key roles in immunomodulation, autophagy, stress resistance, pro-longevity, and telomere protection. Our findings also indicate that MDSPC transplantation increased proteoglycan content by regulating chondrocyte proliferation.

**Conclusions:**

Together, these findings demonstrate the ability of systemically transplanted young MDSPCs to preserve a healthy homeostasis and promote tissue regeneration at the molecular and tissue level in progeroid AC. These results highlight the therapeutic potential of systemically delivered multipotent adult stem cells to prevent age-associated AC degeneration.

**Supplementary Information:**

The online version contains supplementary material available at 10.1186/s12979-021-00239-8.

## Background

Ageing-related degeneration of the knee articular surface, degradation of aggrecans, and loss of matrix tensile strength and stiffness [1, 2] results in osteoarthritis (OA), the most prevalent and debilitating joint disease of advanced age and the leading cause of disability in older adults worldwide [3, 4]. Ageing results in a disruption of cartilage homeostasis. Accumulation of catabolic factors—such as pro-inflammatory cytokines, senescence-associated secretory phenotype (SASP) factors [5], and matrix metalloproteinases (MMPs) alters the tissue microenvironment, contributes to oxidative stress, and can augment inflammatory responses [6, 7]. With increasing age, chondrocyte density and responsiveness to proliferative and anabolic factors are reduced [8, 9]. Because articular chondrocytes rely on autophagy as the primary mechanism for maintaining healthy function and survival [10], the gradual decrease of chondrocyte autophagic activity during ageing induces senescence, ultimately increasing OA severity [11].

While tissue- and cellular-level changes in aged cartilage have been characterized [12], an effective therapy or preventative treatment for age-related articular cartilage (AC) degeneration has not been developed. Current cellular treatments, including autologous chondrocyte implantation, autologous matrix-induced chondrogenesis (AMIC), and intra-articular injection of mesenchymal stem cells, have been effective for small, mainly injury-induced articular cartilage loss [13–16]; however, their effectiveness is questionable in cases of cartilage degradation, rheumatic disease, and considerable restriction of joint mobility, which substantially limits their applicability for the treatment of ageing-related changes [17].

We have isolated a unique population of adult multipotent stem cells, muscle-derived stem/progenitor cells (MDSPCs), from skeletal muscle via a modified preplate technique [18]. MDSPCs have the capacity for long-term proliferation, self-renewal, and multi-lineage differentiation, and are resistant to oxidative stress, all of which likely contribute to their ability to promote regeneration [19–23]. MDSPCs have also been shown to undergo chondrogenic differentiation in vitro and to repair cartilage defects as efficiently as chondrocytes [23], after local transplantation in a young host, injury-induced OA model [24]. Previously, we established that MDSPCs isolated from naturally aged and progeroid (i.e., accelerated ageing) mice showed a reduction in stemness capacity, including proliferation and differentiation; however, these phenotypic impairments were rescued by co-culture with, or by conditioned media from young MDSPCs [22, 25]. In addition, we previously demonstrated that systemic transplantation of young MDSPCs into a progeroid mouse model delayed the onset of multiple age-related pathologies, leading to a doubling of lifespan and a significant extension of healthspan [22]. Our recently published results indicate that systemic transplantation of young MDSPCs into naturally aged (2-yr old) mice restores aging AC—at the molecular, tissue, and functional levels—highlighting their therapeutic ability to reverse age-related osteoarthritis [26]. Together, these observations strongly suggest that MDSPCs exert a therapeutic effect on the aged microenvironment by systemically-acting secreted factors.

*Zmpste24*^*−/−*^ mice are an established murine model of Hutchinson-Gilford progeria syndrome (HGPS), which features many musculoskeletal degenerative changes similar to those of advanced ageing [27–30]. HGPS is manifested predominantly in the connective tissue, with the most prominent histological changes observed in the cartilage, bone, skin, and cardiovascular tissues [31]. Here, we report that systemic transplantation of MDSPCs rejuvenates progeroid AC at the molecular and tissue levels in *Zmpste24*^*−/−*^ mice.

## Results

### Articular cartilage of progeroid mice shows ageing-related imbalances associated with local catabolic and anabolic activity

**Fig. 1:**
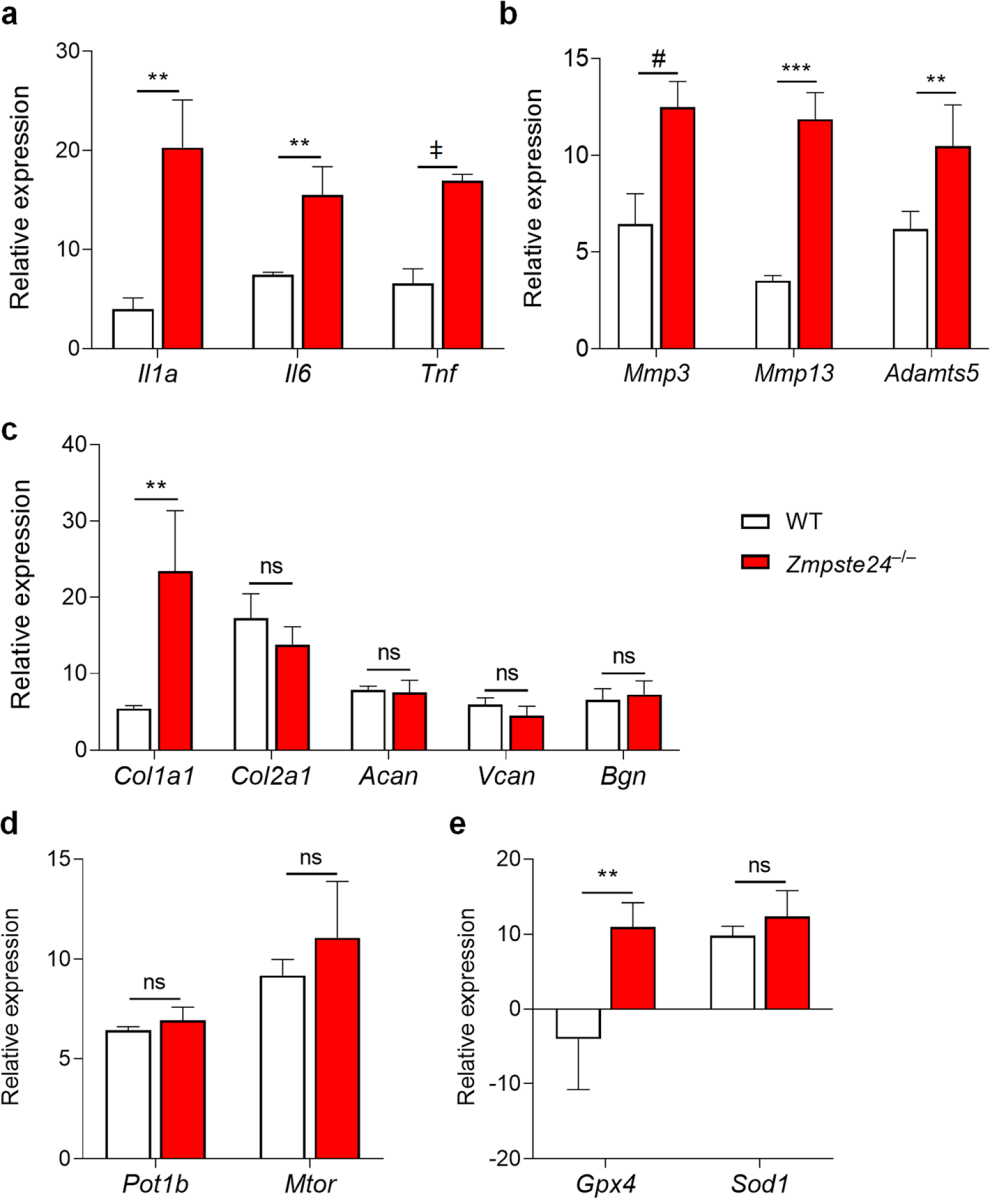
Alteration in Gene Expression Profile of Articular Cartilage in Zmpste24-deficient (*Zmpste24*^−/−^) Progeroid Mice. Articular cartilage from 5–6 month-old adult wild type (*n* = 6) and age-matched *Zmpste24*^−/−^ littermate knee joints (*n* = 5) were analyzed for the mRNA expression level of (**a-c** genes encoding pro-inflammatory cytokines and senescence-associated secretory phenotype (SASP) factors (*Il1a*, *Il6*, and *Tnf*), extracellular matrix (ECM) proteinases (*Mmp3*, *Mmp13*, and *Adamts5*), ECM components (*Col1a1*, *Col2a1*, *Acan*, *Vcan*, and *Bgn*) using qRT-PCR. **d-e** Genes associated with telomere protection (*Pot1b*), autophagy suppression (*Mtor*), antioxidant response (*Gpx4*), and oxidative stress-response (*Sod1*). Expression values are relative to housekeeping genes, *Gapdh and Hmgb1.* Data are mean ± SEM. **p < 0.01, ***p < 0.001, ^#^*p* < 0.0001, ^ǂ^*p* < 0.000001, ns: not significant using two-tailed, unpaired Student’s *t*-test or Welch’s unequal variance *t*-test

To identify age-associated articular cartilage (AC) changes in progeroid mice, gene expression in knee joints of 5–6 month-old *Zmpste24*^*−/−*^ mice was compared to that of age-matched wild-type (WT) littermates. Quantitative reverse transcriptase polymerase chain reaction (qRT-PCR) was used to assess expression of genes encoding pro-inflammatory cytokines, extracellular matrix (ECM) proteinases, and ECM components (Fig. 1). In the AC of progeroid mice, we observed a statistically significant increase in *Il1a*, *Il6*, and *Tnf*—pro-inflammatory factors associated with the senescence-associated secretory phenotype (SASP)—compared to WT littermates (Fig. 1a). Gene expression of *Mmp3*, *Mmp13*, and *Adamts5*—catabolic factors involved with cleaving ECM components such as collagen type II and aggrecans, [32–34] were also significantly increased in *Zmpste24*^−/−^ progeroid mice (Fig. 1b). In addition, we observed a significant increase in the expression level of *Col1a1*, responsible for cartilage tensile strength and stiffness, in the articular cartilage of *Zmpste24*^−/−^ progeroid mice compared to WT littermates; however, the expression level of pro-regenerative matrix cellular components such as *Col2a1*, *Acan, Vcan, and Bgn* were not significantly altered (Fig. 1c). Of note, we did not observe a significant difference in telomere protection (*Pot1b*) or autophagy suppression (*Mtor*) genes in *Zmpste24*^−/−^ progeroid mice compared to WT mice (Fig. 1d). Furthermore, the antioxidant response gene (*Gpx*) was significantly increased in progeroid mice compared to WT mice, while no change was observed in oxidative stress-response (*Sod1*) expression. (Fig. 1e). Taken together, these findings demonstrate a catabolic-anabolic imbalance in the AC of progeroid *Zmpste24*^−/−^ mice.

### Systemic transplantation of young MDSPCs preserves healthy articular cartilage homeostasis in progeroid mice

**Fig. 2:**
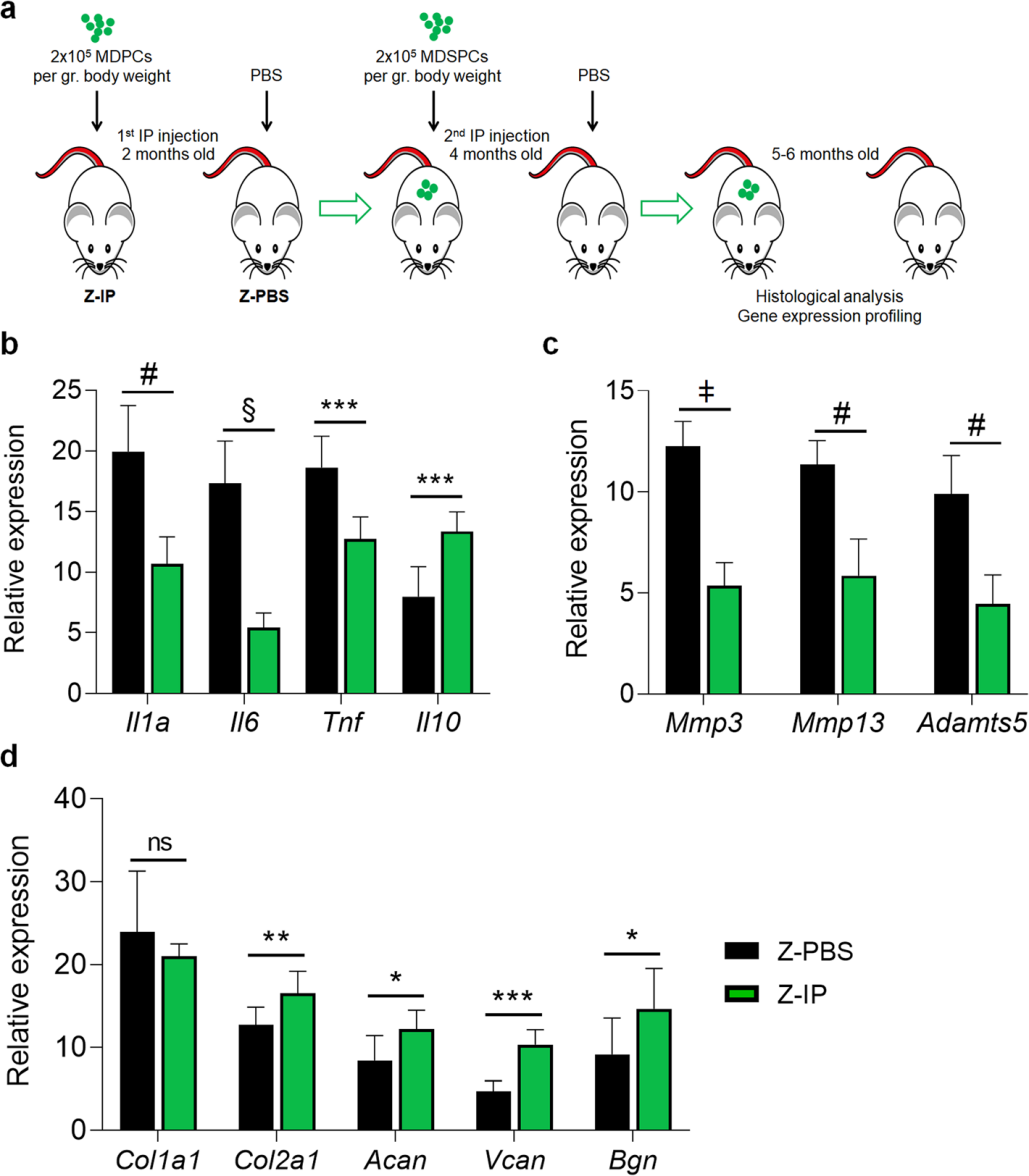
Effect of Young MDSPC Systemic Transplantation in the *Zmpste24*^−/−^ Progeroid Mouse Articular Cartilage Microenvironment. **a** Schematic illustrating the experimental design. **b-d** Quantitation of pro-inflammatory cytokines and SASP markers (*Il1a*, *Il6*, and *Tnf*) and anti-inflammatory cytokine (*Il10*), extracellular matrix (ECM) proteinases (*Mmp3*, *Mmp13*, and *Adamts5*), and ECM components (*Col1a1*, *Col2a1*, *Acan*, *Vcan*, and *Bgn*) relative mRNA levels, measured by qRT-PCR from the knee articular cartilage of *Zmpste24*^−/−^ mice intraperitoneally (IP) injected with MDSPCs (Z-IP, *n* = 7) or PBS (Z-PBS, *n* = 9). Expression values are relative to housekeeping genes, *Gapdh and Hmgb1.* Data are mean ± SEM. *p < 0.05, **p < 0.01, ***p < 0.001, ^#^*p* < 0.0001, ^§^*p* < 0.00001, ^ǂ^*p* < 0.000001, ns: not significant using two-tailed, unpaired Student’s *t*-test or Welch’s unequal variance *t*-test

To determine whether the loss of AC homeostasis due to accelerated ageing in *Zmpste24*^−/−^ mice can be prevented, we investigated the effect of systemic transplantation of young MDSPCs on the AC microenvironment. Littermate pairs of *Zmpste24*^−/−^ mice were injected intraperitoneally (IP) with 2 × 10^5^ young MDSPCs per gram of body weight (Z-IP) or an equal volume of PBS (Z-PBS) at 2 months of age and again at 4 months of age (Fig. 2a). The AC from the knee joints was processed at 5–6 months of age and gene expression was measured using qRT-PCR. The expression of genes associated with pro-inflammatory cytokines and SASP factors, including *Il1a*, *Il6*, and *Tnf*, which are upregulated during ageing and enhance matrix degradation [35], were significantly downregulated in Z-IP articular cartilage compared to Z-PBS littermates (Fig. 2b). Importantly, the expression of *Il10*, an immunomodulatory and anti-inflammatory cytokine, was significantly increased in Z-IP mice (Fig. 2b). Gene expression of *Mmp3*, *Mmp13, and Adamts*5 were also significantly decreased in articular cartilage of Z-IP mice compared with Z-PBS mice (Fig. 2c). Moreover, Z-IP mice showed significant increases in expression of genes responsible for cartilage mechanical support and tissue repair (*Col2a1*), cartilage resilience (*Acan*), chondrocyte proliferation and differentiation (*Vcan*), and cartilage growth (*Bgn*) compared to Z-PBS mice (Fig. 2d) [36–39]. Of note, we did not observe a difference in *Col1a1* gene expression between treatment groups. These data provide evidence that MDSPC transplantation inhibited genes that promote cartilage degradation while activating pro-regenerative genes, thereby preserving a healthy anabolic-catabolic balance in progeroid AC.

### Systemic transplantation of young MDSPCs alters expression of ageing-related genes in articular cartilage of progeroid mice

**Fig. 3:**
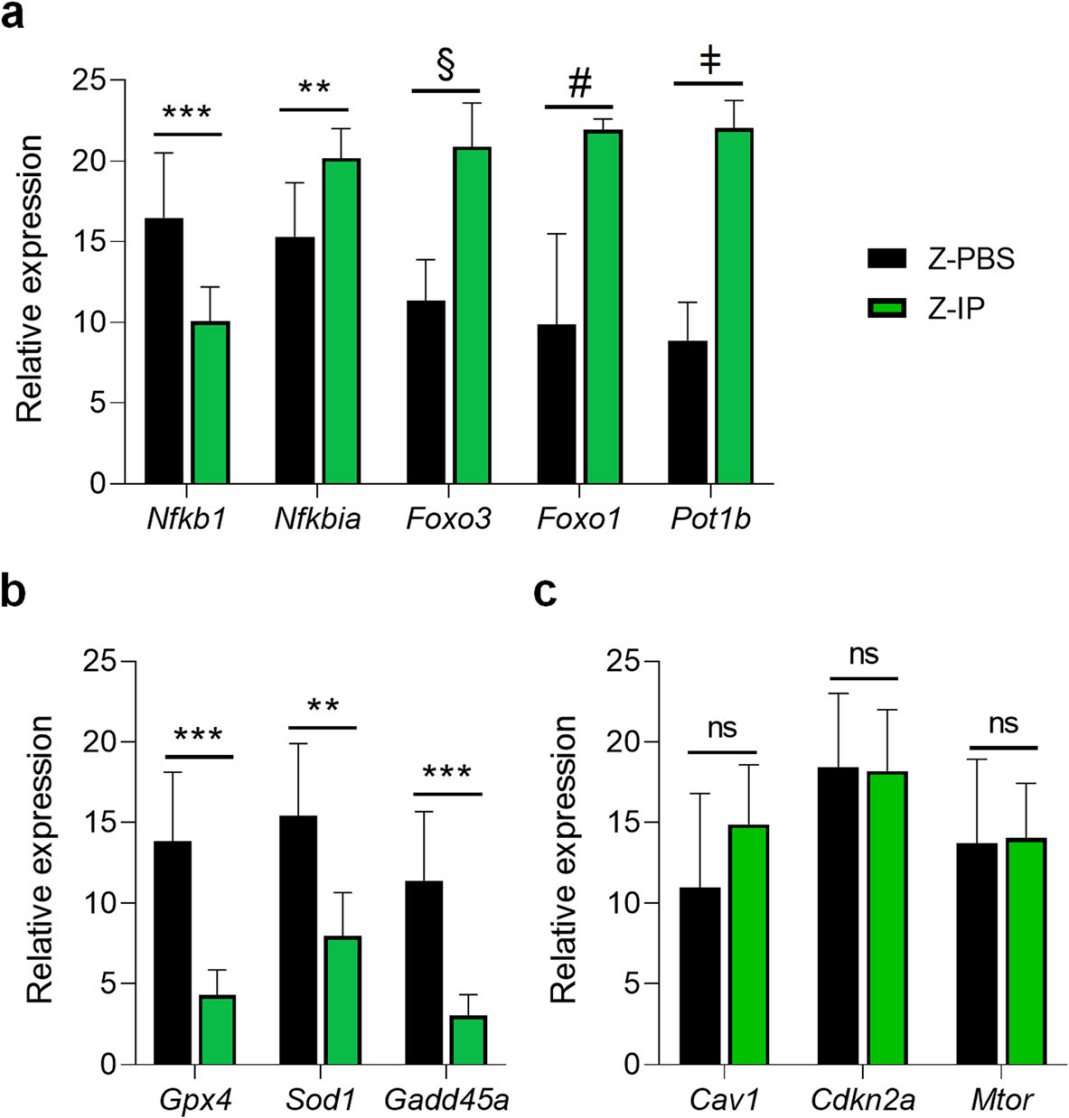
Systemic Transplantation of young MDSPCs Promotes a Healthy Cartilage Homeostasis in *Zmpste24*^−/−^ Progeroid Mice. Relative mRNA levels were measured by qRT-PCR from the knee articular cartilage of *Zmpste24*^−/−^ mice intraperitoneally (IP) injected with MDSPCs (Z-IP, *n* = 7) or PBS (Z-PBS, *n* = 9) and analyzed for the presence of (**a**) a key ageing signaling pathway mediator (*Nfkb1*), its inhibitor (*Nfkbia*), autophagy and pro-longevity markers (*Foxo1 and Foxo3*), and telomere protection (*Pot1b*), (**b**) antioxidant response (*Gpx4*), oxidative stress-response (*Sod1*), and growth arrest and DNA damage response (*Gadd45a)*, (**c**) endocytosis and stress-induced senescence (*Cav1*), the cellular senescence/tumor suppressor mechanism (*Cdkn2a*), and the mammalian target of rapamycin (*Mtor*) genes. Expression values are relative to housekeeping genes, *Gapdh and Hmbg1.* Data are mean ± SEM. **p < 0.01, ***p < 0.001, ^#^*p* < 0.0001, ^§^*p* < 0.00001, ^ǂ^*p* < 0.000001, ns: not significant using two-tailed, unpaired Student’s *t*-test or Welch’s unequal variance *t*-test (a-c and e-g)

We next investigated whether MDSPC transplantation can prevent the changes in expression of key ageing-related genes in the AC of *Zmpste24*^−/−^ progeroid mice (Fig. 3). Our results demonstrate that MDSPC transplantation significantly down-regulated *Nfkb1*, the master regulator and transcription factor most highly correlated with ageing. Interestingly, the expression level of the NF-κB inhibitor, *Nfkbia*, was significantly upregulated in Z-IP mice (Fig. 3a) compared to Z-PBS littermates. Our results also revealed a significant increase in the FoxO transcription factors, *Foxo3* and *Foxo1*, in the AC of Z-IP mice compared to Z-PBS mice (Fig. 3a). With age, chondrocytes exhibit senescent phenotypes, including telomere shortening [40]. Notably, the telomere protection gene *Pot1b*, known as a pro-longevity marker, [41] was robustly increased in the cartilage of Z*-*IP mice (Fig. 3a). MDSPC transplantation significantly decreased the expression level of the oxidative stress-response gene *Sod1*, the antioxidant enzyme glutathione peroxidase 4 (*Gpx4*), and the growth arrest and DNA damage response gene *Gadd45a*, compared to Z-PBS mice (Fig. 3b). We did not detect any differences in *Mtor*—the key regulator and suppressor of autophagy during ageing (Fig. 3c). Also, the expression level of stress-induced senescence gene *Cav1* [42, 43]—a major component of the caveolae structure and a transmembrane protein, and of cellular senescence gene *Cdkn2a*, also known as p16 [44], did not change following MDSPC transplantation (Fig. 3c). Together, these results indicate systemic transplantation of young MDSPCs restores aged articular cartilage homeostasis and improves maintenance at a molecular level.

### Systemic transplantation of young MDSPCs rejuvenates articular cartilage in *Zmpste24*^−/−^ progeroid mice

**Fig. 4:**
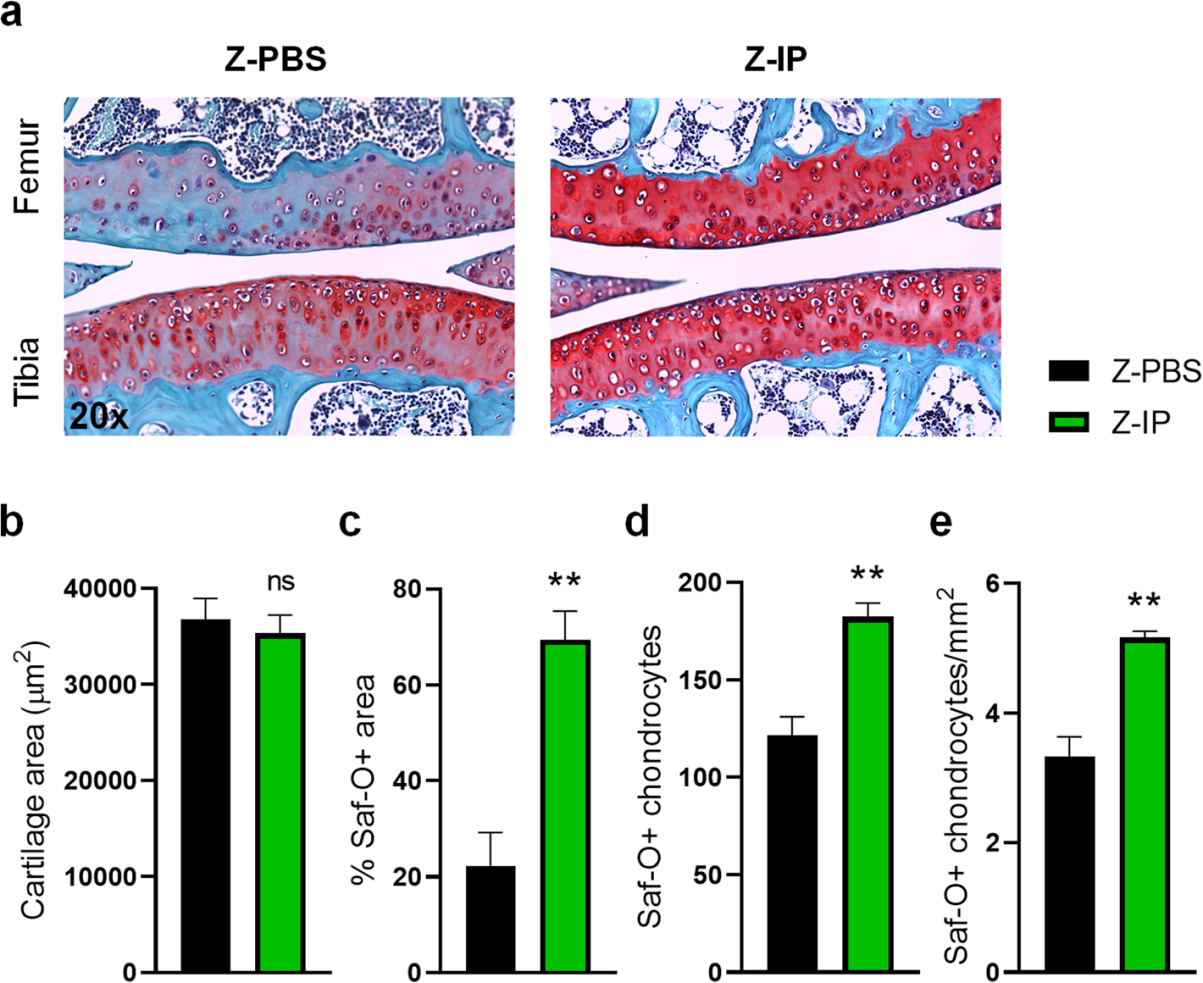
Histopathology of Articular Cartilage Following Systemic Transplantation of Young MDSPCs in *Zmpste24*^−/−^ Mice. **a** Representative images (20x magnification) of Safranin-O (Saf-O) stained femoral condyle and tibial plateau from PBS- (Z-PBS, *n* = 3) and MDSPC-intraperitoneally transplanted (Z-IP; *n* = 3) *Zmpste24*^−/−^ mice at 5–6 months of age. Quantitative histomorphometric analysis shows (**b**) total cartilage area, (**c**) percent of Saf-O + cartilage area, (**d**) chondrocyte number, and (**e**) chondrocyte density. Data are mean ± SEM. *p < 0.05 with two-tailed, unpaired Student’s *t*-test

To determine whether MDSPC treatment maintains healthy AC tissue structure by preventing the loss of ECM proteoglycans, knee joints were stained with Safranin-O (Saf-O). Saf-O staining in the femoral condyle and tibial plateau (Fig. 4a) of the articular cartilage revealed striking changes between Z-IP and Z-PBS mice. In fact, while both groups maintained a similar overall total cartilage area (Fig. 4b), 69 % of the cartilage area in Z-IP mice was Saf-O + compared to only 23 % in Z-PBS mice (Fig. 4c), indicating proteoglycan enrichment after MDSPC transplantation. The knee joints of Z-IP mice also had a greater number of chondrocytes and density compared to Z-PBS control mice (Fig. 4d and e). Together, these findings strongly suggest that MDSPC treatment confers protection against the loss of AC matrix, in part by increasing cartilage cellularity and preventing age-associated degradation.

To investigate how young MDSPCs rejuvenate aged AC, we analyzed the AC throughout the knee joint for donor cell engraftment. MDSPCs were transduced with a retroviral vector containing a nuclear localization sequence (*nLacZ*) prior to transplantation. As a positive control for tracking donor cells, transduced MDSPCs were also intramuscularly (IM) transplanted into gastrocnemius muscles of WT control mice. Large numbers of *LacZ* + MDSPCs were detected in the control IM injected muscle, however, no donor MDSPCs were detected in the AC—or surrounding tissues—of any Z-IP mouse (Supplementary Fig. S1). This indicates that the beneficial effects are not due to direct tissue or chondrocyte reconstitution by donor MDSPCs, but are rather mediated through their therapeutic secreted factors that act systemically.

## Discussion

Given that AC degeneration causes OA, a significant cause of disability worldwide, we sought to identify age-related changes in the cartilage microenvironment in a mouse model of accelerated ageing. Furthermore, we evaluated an intervention to delay or reverse the degenerative effects of accelerated ageing. Here, we report, for the first time, the prevention of progeroid AC degeneration—at the molecular and tissue levels—by systemic transplantation of young WT MDSPCs.

*Zmpste24*^−/−^ mice exhibit gene expression signatures robustly associated with ageing in AC—such as increased SASP factors including pro-inflammatory cytokines (*II1a* and *Il6*) and catabolic matrix-degrading proteinases (*Mmp3*, *Mmp13*, and *Adamts5*), which further drive age-associated pathologies such as hypertrophic chondrocyte differentiation. However, after MDSPC treatment, *Zmpste24*^−/−^ gene expression profiles remained similar to those of WT littermates [12, 37]. Furthermore, the immunomodulating and anti-inflammatory cytokine *Il10* and an ECM component that plays an important role in tissue remodeling and repair, *Col2a1* [38], were upregulated in the AC of Z-IP mice. Rejuvenation was also supported by increased expression of vital matrix proteoglycans *Acan, Vcan, and Bgn*, which are responsible for cartilage tensile strength, resilience, and cartilage growth. Together, these results demonstrate the restoration of progeroid cartilage to a “healthy” state.

The fact that systemic transplantation provides such a remarkable improvement in progeroid AC, despite the fact that no donor cells could be detected in AC, reinforces our hypothesis that the observed effects are induced via secreted paracrine/endocrine factors. This is consistent with our previous reports [22, 25] where young (but not old) MDSPCs restored the dysfunction of progeroid and naturally aged MDSPCs when co-cultured in vitro, and that transplantation of young MDSPCs extended both the lifespan and healthspan of progeroid mice through systemically acting secreted factors. Indeed, more recently, we have also shown that systemic therapy with young MDSPCs results in restoration of naturally aged (2-yr old) articular cartilage—at the molecular, tissue, and functional levels [26]. Similarly, no transplanted cells were found in AC, however, systemic transplantation of MDSPC reduced inflammatory factors and ECM proteinases, increased anabolic factors and proteoglycan content, and induced the expression of pro-regenerative and pro-longevity genes. As a result, the MDSPC-treated naturally aged mice exhibited AC regeneration and significant functional improvement [26]. The underlying mechanism(s) of action for this rejuvenation still remains unknown, but our ongoing studies focus on the mechanistic basis of MDSPC systemic effects. We hypothesize that MDSPCs promote the maintenance of healthy AC by secreting chondrocyte-protecting cytokines/growth factors, thereby regulating chondrocyte viability and sensitivity to extrinsic factors and reducing articular cartilage degeneration. In fact, MDSPCs are known to perform a variety of biological functions, including immune regulation, angiogenesis, anti-apoptosis, anti-oxidant, cell homing, and promotion of tissue-specific cell differentiation [45]. Therefore, it is not far-fetched to suggest that pro-regenerative factors secreted by MDSPCs preserves the local AC microenvironment in progeroid mice by increasing anabolic activity while simultaneously inhibiting catabolic activity.

The fact that the DNA damage-inducible and oxidative stress genes *Gadd45a*, *Sod1*, *Gpx4*, were all significantly decreased following systemic transplantation, indicates that MDSPCs may also preserve AC through cyto-protective mechanisms. This is supported by the observation that we measured a profound increase in expression of the telomere protection gene, *Pot1b*, demonstrating a protective effect of MDSPCs on chromosomal stability. In fact, recent studies have shown that maintenance of telomere length can reverse neurodegeneration, delay metabolic ageing in mice, and increase lifespan [41, 46].

The transcription factor NF-κB, a central controller and mediator of ageing-related signaling pathways, regulates many immune responses [47] and is also a candidate activator of many ageing-related transcriptional changes in human and mouse tissues, particularly stress-inducing stimuli such as pro-inflammatory factors and SASPs [48]. Previous studies by Robbins et al. indicate that *Nfkb1* transcriptional activity is up-regulated in a variety of tissues in both natural ageing and in mouse models of human progeria caused by defective DNA damage repair mechanisms [49]. Skin-derived human fibroblasts from HGPS patients and aged individuals also show increased inflammatory gene expression and increased levels of NF-κB activation compared to cells from young individuals [48, 50]. Similar to previous reports [51], systemic transplantation of MDSPCs results in significant reduction of *Nfkb1* expression and an increase in expression of the *Nfkb1* inhibitor, *Nfkbia*. Further studies are needed to investigate the mechanism(s) underlying modulation of NF-kB and other inflammatory factors.

Defective autophagy is a key feature of age-related diseases, and recent observations indicate that this process is compromised in ageing cartilage [52, 53] through FoxO downregulation [54]. FoxO transcription factors, which have recently been found to play vital roles in postnatal cartilage development, maturation, homeostasis, and protection against OA-associated cartilage damage [54] as well as to protect against cellular ageing and to enhance autophagy, are suppressed in human OA and mouse cartilage [11, 55]. *Foxo1* and *Foxo3* regulate chondrocyte proliferation and differentiation [54] and their expression levels are reduced in both ageing and OA-affected cartilage in humans and mice [54]. In our study, the prominent upregulation of *Foxo1* and *Foxo3* in AC in MDSPC-treated mice suggests that our cellular therapy prevents chondrocyte ageing and delays the onset of OA-like symptoms, in part, through these mechanisms. It appears that, by preserving cartilage cellularity, the bioactive paracrine/endocrine factors secreted by MDSPCs had beneficial effects on the aged cartilage tissue microenvironment, leading to more favorable conditions for tissue regeneration. This results in increased chondrocyte density and an enrichment of ECM proteoglycans, thereby maintaining more normal knee joint histology.

Collectively, our results suggest that the mechanisms behind AC rejuvenation in progeroid mice are in part due to activation of telomere protection mechanisms, modulation of the inflammatory SASP cascade, and maintenance of pro-regenerative and pro-longevity pathways. Further studies will identify MDSPC secretomes, specifically those which are activated after IP transplantation. Limitations of this study include that we did not assess functional effects of MDSPC transplantation or possible effects of sex differences between donor and host. Future studies will investigate if the observed restoration of AC at the molecular and cellular levels after MDSPC systemic transplantation lead to functional stability or improvement in progeroid mice.

## Conclusions

These results demonstrate that treating a mouse model of accelerated ageing with a systemic injection of young multipotent adult stem cells prevents age-related cartilage degradation and maintains the catabolic/anabolic balance in host tissues. This cellular treatment promotes a “healthy” homeostasis and stimulates tissue regeneration. Young MDSPCs, and/or their secreted factors, thus represent a potential novel therapeutic strategy for preventing or treating age-related AC degeneration.

## Methods

### MDSPC isolation

Young WT MDSPCs were isolated from the hindlimb skeletal muscle of 21 day-old female mice via a modified preplate technique [18]. MDSPCs were cultured and expanded for transplantation in proliferation medium (PM): Hi-glucose DMEM supplemented with 10 % horse serum, 10 % fetal-bovine serum (FBS), 1 % penicillin-streptomycin (all from Invitrogen), and 0.5 % chick embryo extract (CEE, Accurate Chemical). Cells are cultured on collagen type I-coated flasks.

### Animal Husbandry

All animal experiments were performed with the approval of the Northwestern University Institutional Animal Care and Use Committee. 17-day-old mice are ear tagged and tail snips are collected and mailed through the Northwestern University in-house system to Transnetyx for genotyping. Results are received within 72 h with over 99.9 % accuracy. Primers and PCR conditions for genotyping *Zmpste24*^−/−^ mice have been previously published [30]. Mice were maintained in a pathogen-free facility at 23–24 °C under a 12-hr light, 12-hr dark regimen and fed ad libitum a standard chow which is gamma-irradiated. Mice were always studied in sibling pairs to minimize environmental variables. Only if breeding produces two or more *Zmpste24*^−/−^ mice would the litter be used in an exposure study so that one mutant animal is treated with the experimental treatment and the other receives vehicle only.

### Cell Transplantation

MDSPCs suspended in 50 µL of buffered saline (PBS) were transplanted via intraperitoneal (IP) administration into 4-5-monthold *Zmpste24*^−/−^ mice at 2 × 10^5^ MDSPCs per gram body-weight. A littermate mutant animal was injected with vehicle only (PBS). The injection was repeated 2 months later. Mice were sacrificed at 5–6 months of age and the right hind limbs were harvested for histopathological analysis and the left knee joint were isolated for gene expression analysis. Both male and female mice were included in the transplantation study.

### Histology

Right hind limbs were fixed in PFA for 2 days, stored in PBS at 4^°^C overnight, and then paraffin embedded. Sections were cut to 4 μm, collected at 100 μm intervals, and stained with Safranin O (Saf-O)−Fast Green as described previously to evaluate the proteoglycan content and pathological changes such as cartilage degradation [56]. To detect *nLacZ* expression, paraffin embedded sections of AC were deparaffinized in serial washes of xylene and rehydrated in sequential washes of 100–70 % ethanol. The tissue sections were then incubated with X-gal solution overnight at room temperature and counterstained with eosin.

### Histomorphometry

Histomorphometric analyses were performed using NIS-Elements software (Nikon, AR 5.11.03). The Saf-O + area was measured impartially by the NIS software using identical thresholding parameters between all images (pixels falling below the threshold intensity of 20 were uncounted) by detecting the total red (Saf-O+) pixel area. The total cartilage area was manually selected with border detection assistance using the NIS software’s thresholding tools. Saf-O + chondrocytes were manually counted for each femoral condyle and tibial plateau and graphed as average Saf-O + chondrocytes and number of Saf-O + chondrocytes per mm^2^ of total articular cartilage. Chondrocytes were considered Saf-O + if they were surrounded by red-stained matrix.

### RNA isolation and qPCR

To measure mRNA expression, total RNA was extracted from articular cartilage isolated from paraformaldehyde (PFA)-fixed knee joints using an RNeasy® mini kit for formalin-fixed, paraffin-embedded (FFPE) tissue (Qiagen) according to manufacturer’s protocol. RNA quality was validated using an Eppendorf Bio-Spectrophotometer, and 100 ng of total RNA was reverse-transcribed according to the manufacture’s protocol using iScript Advanced cDNA synthesis kit (Bio-Rad Laboratories). Pre-amplification of the primers was carried out according to the manufacturer’s protocol using a Pre-amplification kit (Bio-Rad Laboratories). The pre-amplified cDNA was diluted and used for analysis of gene expression changes in 10 µL reactions using SYBR green advanced master mix kit (Bio-Rad Laboratories) and the gene of interest primer pairs. Data were analyzed with the ΔCt method and gene expression was normalized to average expression of housekeeping genes, *Gapdh* and *Hmgb1*. Primers for the genes of interest were obtained from Bio-Rad Laboratories and the primer catalog identification IDs are listed in Table 1.

### Statistics

Statistical analyses were carried out using the SigmaPlot (Jandel Scientific v14.0) software package. The two-tailed unpaired Welch’s unequal variance *t*-test, two-tailed Student *t*-test, or the Mann-Whitney Rank sum test were used where appropriate for direct comparisons between treatment and control groups. All values are expressed as the mean ± SEM., and *p* < 0.05 was considered significant.

**Table 1:** Primer list

Gene name	Traditional classification (Common name)	Catalog identification
*Acan*	Aggrecan	qMmuCED0046843
*Adamts5*	a-disintegrin and metalloproteinase with thrombospondin motif 5	qMmuCED0045481
*Bgn*	Biglycan	qMmuCED0046901
*Cav1*	Caveolin 1	qMmuCID0020997
*Cdkn2a*	Cyclin dependent kinase inhibitor 2a	qMmuCED0038108
*Col1a1*	Collagen type I alpha 1	qMuCED0044222
*Col2a1*	Collagen type 2 a1	qMuCID0006546
*Col10a1*	Collagen type 10 alpa1	qMuCID0008115
*Foxo1*	Fork head box transcription factor 1	qMmuCID0016391
*Foxo3*	Fork head box transcription factor 3	qMmuCED0004522
*Gadd45a*	Growth arrest and DNA damage inducible alpha	qMmuCED0001074
*Gapdh*	Glyceraldehyde 3-phosphate dehydrogenase	qMmuCED0027497
*Gpx4*	Glutathione peroxidase	qMmuCED0001062
*Hmgb1*	High mobility group box 1	qMmuCE00041193
*Il1a*	Interleukin 1 alpha	qMmuCID005637
*Il6*	Interleukin 6	qMmuCED0045760
*Il10*	Interleukin 10	qMmuCED0044967
*Mmp3*	Matrix metalloproteinase 3	qMmuCED0049170
*Mmp13*	Matrix metalloproteinase 13	qMmuCED0050490
*Mtor*	Mammalian target of rapamycin	qMmuCED0047795
*Nfkb1*	Nuclear factor kappa b1	qMmuCID0005357
*Nfkbia*	Nuclear factor kappa b inhibitor	qMmuCED0045043
*Pot1b*	Protection of telomeres 1b	qMmuCED0045942
*Sod1*	Superoxide dismutase 1	qMmuCID0026086
*Tnf*	Tumor necrosis factor	qMmuCED004141
*Vcan*	Versican	qMmuCED0046650

## Supplementary Information

Additional file 1: **Supplementary Figure S1.** Determination of donor cell engraftment after transplantation of *Zmpste24*^-/-^ mice with young MDSPCs. Articular cartilage of *Zmpste24*^-/-^ mice (Z-IP) intraperitoneally injected with 2 x 10^5^ MDSPCs transduced to express a nuclear *LacZ*reporter gene for donor cell tracking, were stained with X-gal and Eosin (pink) to determine sites of engraftment. Gastrocnemius muscles of 6-month old wildtype (WT) mice intramuscularly (IM) injected with the same MDSPCs were identically stained as a positive control. Brightfield images show donor cells (*LacZ*+, blue) were not detected in articular cartilage, however, as expected, large numbers of donor cells were detected in gastrocnemius muscles of control IM injected WT mice (top row seen at 5x magnification and bottom row seen at 10x magnification).

## Data Availability

The datasets used and/or analyzed during the current study are available from the corresponding author on reasonable request.
