# Supplementary material for: Systemic transplantation of adult multipotent stem cells prevents articular cartilage degeneration in a mouse model of accelerated ageing
Source: Immun Ageing. 2021 Jun 7;18:27. doi: 10.1186/s12979-021-00239-8 (PMC8183038; doi:10.1186/s12979-021-00239-8)
Supplement: Supplementary file 1 — Supplementary Figure S1. Determination of donor cell engraftment after transplantation of Zmpste24-/- mice with young MDSPCs. Articular cartilage of Zmpste24-/- mice (Z-IP) intraperitoneally injected with 2 x 10^5^ MDSPCs transduced to express a nuclear LacZreporter gene for donor cell tracking, were stained with X-gal and Eosin (pink) to determine sites of engraftment. Gastrocnemius muscles of 6-month old wildtype (WT) mice intramuscularly (IM) injected with the same MDSPCs were identically stained as a positive control. Brightfield images show donor cells (LacZ+, blue) were not detected in articular cartilage, however, as expected, large numbers of donor cells were detected in gastrocnemius muscles of control IM injected WT mice (top row seen at 5x magnification and bottom row seen at 10x magnification). [file 12979_2021_239_MOESM1_ESM.docx]

SUPPLEMENTARY INFORMATION

**Systemic Transplantation of Adult Multipotent Stem Cells Prevents Articular Cartilage Degeneration in a Mouse Model of Accelerated Ageing**

Seth D. Thompson, Rajeswari Pichika, Richard L. Lieber, Mitra Lavasani

**Supplementary Figure S1.** Determination of donor cell engraftment after transplantation of *Zmpste24^−/−^* mice with young MDSPCs. Articular cartilage of *Zmpste24^−/−^* mice (Z-IP) intraperitoneally injected with 2 x 10^5^ MDSPCs transduced to express a nuclear *LacZ* reporter gene for donor cell tracking, were stained with X-gal and Eosin (pink) to determine sites of engraftment. Gastrocnemius muscles of 6-month old wildtype (WT) mice intramuscularly (IM) injected with the same MDSPCs were identically stained as a positive control. Brightfield images show donor cells (*LacZ*+, blue) were not detected in articular cartilage, however, as expected, large numbers of donor cells were detected in gastrocnemius muscles of control IM injected WT mice (top row seen at 5x magnification and bottom row seen at 10x magnification).

**Z-IP (Articular cartilage) WT IM Control (Muscle)**


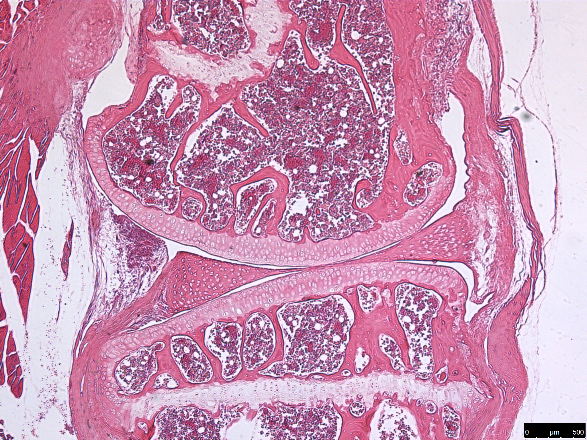

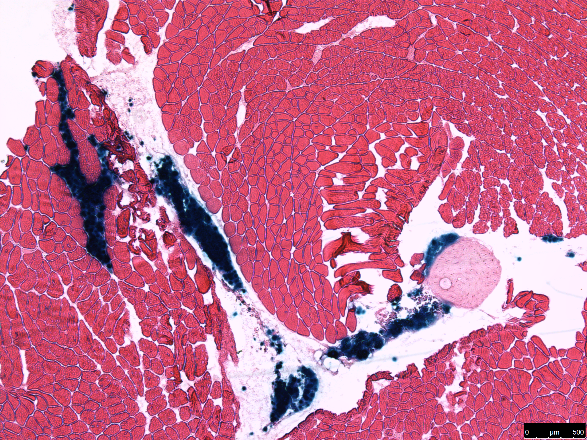
 **5x**


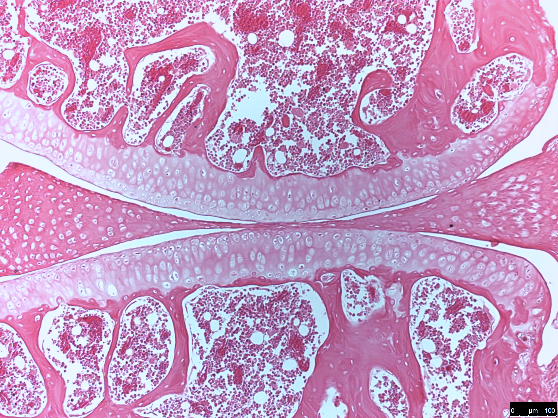

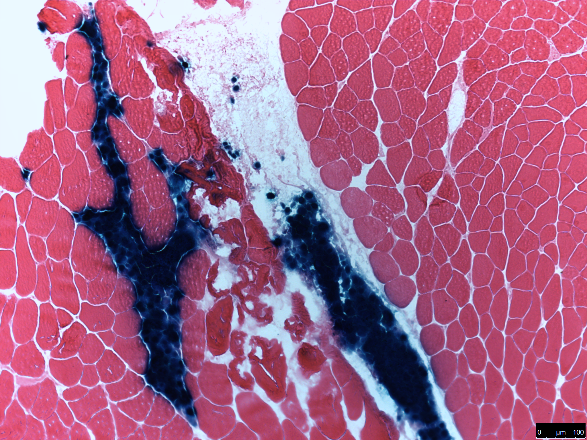
 **10x**
